# Items of Interest

**Published:** 1896-09

**Authors:** 


### Items of Interest.

---

At the meeting of the American Medical Association at Atlanta, May, 1896, the report of the board of trustees of the American Medical Association for the eight months ending December 31, 1895, was presented as follows:

*Gentlemen*—The trustees beg leave to present the following report for eight months ending December 31, 1895.

This report covers but eight months, owing to the change of the fiscal year:

RECEIPTS.

| | | |
|---|---|---|
| On hand May 1, 1895, | $ 123.96 | |
| Bank collections, | 1,047.42 | |
| Advertisements, | 10,307.67 | |
| Office subscriptions, | 1,280.31 | |
| Reprints, | 1,328.01 | |
| Sales and presswork, | 1,059.65 | |
| Total, | $15,147.02 | |
| Treasurer, | 9,459.93 | |
| | | $24,606.95 |

DISBURSEMENTS.

| | | |
|---|---|---|
| Deposited with treasurer, | $ 557.00 | |
| Pay-roll, mechanical department, | 8,177.94 | |
| Paper | 3,988.61 | |
| Rent | 1,000.00 | |
| Office salaries, | 3,790.00 | |
| Editorial account, | 1,915.89 | |
| Postage, | 1,020.51 | |
| Reporting, | 402.40 | |
| Binding | 184.00 | |
| Commission | 638.85 | |
| Express, | 23.75 | |
| Miscellaneous, insurance, taxes, lighting, etc., | 1,624.17 | |
| Engraving, | 274.79 | |
| | | $23,597.91 |
| Balance on hand, | | $1,009.04 |
| Treasurer had a balance December 31, 1895, of | | 9,075.94 |
| Making a total balance of | | $10,084.98 |

We feel confident you will agree with us that this is a most excellent showing, when we consider the financial condition of the country and the radical change in the policy of the *Journal* instituted at the last meeting. A careful analysis has demonstrated that this policy has reduced the amount which would have been received from advertisements something over $3,000, but we are gratified to report that the measures to secure new members which have been instituted by the trustees, and so ably carried out by the *Journal* editor, have more than made up this deficiency. The members of the Association, May 26, 1894, numbered 4,032 ; May 1, 1896, 4,989. The issue of the *Journal* at the former date was 4,876, at the present, 6,548, making a gain in membership in two years of 950 and requiring an increased issue of 1,672. At present there is due the *Journal* from subscribers $1,400 ; from advertisers, $2,400.69. Since the first of the year the *Journal* office has been moved to the Occidental building, at 61 Market street, an excellent fire-proof building, where it occupies commodious quarters upon the third floor. The previous quarters were dark, did not afford sufficient space for the work, and the property was constantly subject to danger of loss from fire. The present quarters entail an increase in cost for power of nearly $60 per month. A careful study of the situation caused the trustees to feel that this change was a wise as well as a necessary one. Since the beginning of the year it has been necessary to procure an electric motor at a cost of $350, new type at $900, and a folder has been ordered which will cost $1,200. The trustees have under consideration an increase in size of the *Journal*, some eight pages, which will permit a review of the American medical literature as is now done of foreign material, and other important improvements which will add to its value. The trustees feel that it will not be amiss to direct the attention of your body to the fact that all the printing and stationery for the Association is now furnished through the *Journal* office. The expenses of the 1893 meeting were $1,526.85 ; 1894, $2,134.91 ; the 1895, $981.85 ; the greater part of this large reduction in expenses has been due to the changed policy and the more careful supervision of the disbursements.

The improved outlook, the increasing revenue and the lessening need for equipment renders it desirable that steps should be taken to secure permanent quarters. With this object in view, the trustees would respectfully request that permission be granted them to establish a building fund, into which should be paid such portion of the surplus, at the end of each fiscal year, as may be deemed by the trustees wise and expedient ; such sums to be invested until a sufficient amount has been secured to enable them to procure suitable property.

We would suggest that members of the Association who have been upon the list for forty years and over should be entitled to be made honorary members and receive the *Journal* free of cost.

In conclusion, the trustees would express their appreciation of the untiring efforts of the editor and the *Journal* employees to advance its interests. Respectfully submitted,

A. GARCELON, *President*.

E. E. MONTGOMERY,

E. F. INGALS,

J. E. WOODBRIDGE,

JOS. EASTMAN,

J. T. PRIESTLEY,

D. W. GRAHAM,

I. N. LOVE.

The foregoing is a gratifying exhibit of the progress the *Journal* is making.

In this connection it is interesting to note that the *Journal* some time ago, under direction from the association, requested a vote from each member as to what city they favored for the permanent location of the *Journal*. The vote as reported stood as follows: Chicago, 2,128; Washington, 810; New York, 25; Baltimore, 4, with a few scattering votes. This, of course, means that the *Journal* will remain at Chicago, which is its most appropriate home.

---

THERE is a growing disposition among state and local boards of health to improve their efficiency by holding frequent conferences. This is a most commendable plan and cannot fail to prove of great benefit to commonwealths and municipalities. In this connection the following, from the *Atlantic Medical Weekly*, will prove of interest:

Dr. Irving A. Watson, of Concord, secretary of the State Board of Health, has sent a communication to the different boards of health throughout New Hampshire, suggesting that they hold a conference at some time during the present summer. He says:

"In looking at some of the sources through which the public health interests of the state might be materially benefited, it has occurred to our board and also has been suggested by municipal health officers, that a conference of the members of the health departments of our several cities be held at some convenient time and place (perhaps at The Weirs in the latter part of September,) for the purpose of considering questions intimately connected with city health work. We believe that a day might be very profitably devoted to this subject. We shall write all the city health boards a letter similar to this, and if their replies are favorable, a day will be selected and a suitable program arranged. We would also suggest the formation of a permanent organisation of municipal boards of health. Please inform us if this proposed conference and organisation meets with your approval. We also invite suggestions."

Dr. Watson is likewise secretary of the American Public Health Association that meets in Buffalo, September 15–18, 1896, and has shown himself to be a most capable and efficient officer as well as a sanitary expert of distinction.

---

PAJOT, the famous French obstetrician, who has lately died, was about as noted for his wit as for his professional skill. It has been stated, indeed, that his talent for epigram rather hindered than promoted his social success and that it caused his exclusion from the academy. The epitaph that he wrote on Civiale, the inventor of lithotrity, illustrates his penchant for mots, some of which were not so harmless as the following :

For Civiale who's dead and gone
Though sympathetic tears may gush,
Set not upon his grave a stone
Which he would surely rise to crush.

On his study table, amid a curious collection of bric-a-brac, stood a fragment of a shell bearing the following inscription : Presented by His Majesty, the King of Prussia, in my consulting room, January 9–10, 1871.

Pajot in his lectures was sometimes sarcastic against *la belle mere.* "As soon as the child is born," he used to say to his class, "lay it on a table and not on a chair. If you neglect this hint, your patient's heavy mother is likely to come and sit upon it."

Besides the myriad of fellow-creatures whom he ushered into the world, it is asserted that Pajot prevented sixteen desperate mortals from quitting it. A skilful angler, he was wont to spend his nights in a boat under the pont Marie, where would-be suicides often interrupted his sport. For his life-saving exploits he was nicknamed by Tardieu the Newfoundland dog of the faculty.

---

IT IS WELL KNOWN that Dr. Thomas Addis Emmet, of New York, has nearly all his life been a collector of manuscripts, prints and books relating to American history, and it is stated that his valuable collection is to be added to the New York public library, but to be kept together and called the Emmet Library. This will be a most pleasing item of news to all interested in the subject and especially, perhaps, to the Sons and Daughters of the American Revolution, because it makes available for their inspection one of the most instructive and unique collections extant.

